# Supplementary figures and images for: Vitamin B12 Supplementation in Diabetic Neuropathy: A 1-Year, Randomized, Double-Blind, Placebo-Controlled Trial
Source: Nutrients. 2021 Jan 27;13(2):395. doi: 10.3390/nu13020395 (PMC7912007; doi:10.3390/nu13020395)

Figure 1 CONSORT 2010 Flow Diagram

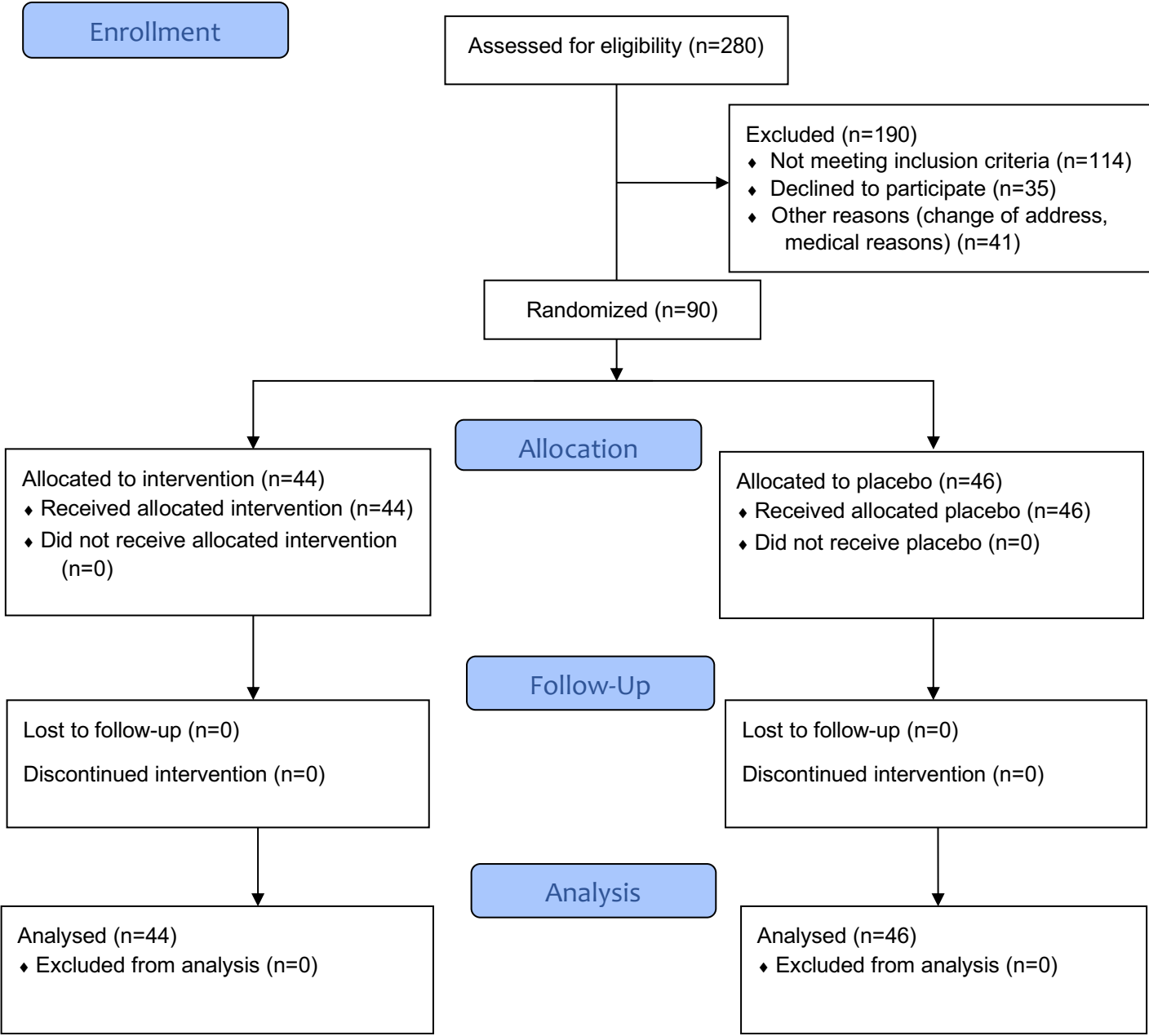

Supplement: Supplementary file 1 [file nutrients-13-00395-s001.pdf]
